# Project over: nocturnal enuresis and urinary disorders

**DOI:** 10.1186/1824-7288-40-S1-A81

**Published:** 2014-08-11

**Authors:** Giuseppe Ragnatela, Marina Picca, Lorenzo Cresta, Mario Fama, Carmela Lo Giudice, Angela Pasinato, Marco Sequi, Pier Luigi Tucci

**Affiliations:** 1 ASL BAT Barletta, Italy; 2 ASL Milano, Italy; 3 ASL Genova, Italy; 4 ASL 17 Monselice-Este, Italy; 5 ASP 6 Palermo, Italy; 6 ASL Vicenza, Italy; 7 ASL 10 Firenze, Italy; 8 Mario Negri Institute for Pharmacological Research, Milan, Italy

## Background

"Enuresis " also known as *bedwetting* or night-time incontinence is the inability to control urination during sleep [1,2]. Mono-symptomatic enuresis (MNE) is the only night-time incontinence and “not mono-symptomatic enuresis” (NMNE) is also the daytime urinary disorders. Bedwetting is a disorder that affects the development of children personality and interferes with social relationships of children and their family.

## Aim

Primary endpoint is to estimate the prevalence of enuresis in patients aged 5 to 14 years, to evaluate the awareness of the problem by the family [3], to assess the discomfort experienced by patients and then to look for the presence of co-factors.

## Methods

The study, promoted by SICuPP (Italian Society of Pediatric Primary Care), was based on a questionnaire filled by the parents of children enrolled by 75 pediatricians from three different regions (Veneto, Tuscany and Puglia).

## Results

We evaluated 3165 children (1618 males and 1547 females). The enuresis is present in 262 children (8.2 % of the study); the prevalence was 16.5% at 5 years 10.5% and 6% NMNE/MNE) and decreases with age up to 4.6% (2.6% and 2% NMNE/ MNE) at the age of 13 years; these data agree with other epidemiological studies . Familiarity and sleep disorders are potential risk factors. Only 33% of parents tell the problem to the pediatrician: 67.8% of the parents don’t speak of it because they consider it unimportant, 2.7% because they are "ashamed", 29.5% for "other reasons". Only 32 patients with enuresis (12.2%) were in treatment. 21.8% of parents think that enuresis "very emotionally involved" their child, 8.5% think that the child is "very limited" in its activities with classmates, 8.7% think that the child is "very concerned" about his health. 33.3% consider bedwetting problem "very important" for the family organization, 24.7% consider that it restricts greatly the activity of the child with classmates, 17.3% is "very concerned" about the health of the child, and finally 11% think that bedwetting is something to be "very ashamed" of.

## Conclusions

Preliminary data show that bedwetting is a "masked" disease. It is important to look for and recognize the problem, to improve the quality of life of patients and their families
